# Impact of county and state immigration policies on immigrant household enrollment in the supplemental nutrition assistance program

**DOI:** 10.1016/j.jmh.2024.100224

**Published:** 2024-02-29

**Authors:** Sofia Argibay, Amy H. Auchincloss, M. Pia Chaparro, Caroline Kravitz, Alexandra Eastus, Brent A. Langellier

**Affiliations:** a Department of Health Management and Policy, Dornsife School of Public Health, Drexel, University. Nesbitt Hall, 3215 Market St., Philadelphia, PA 19104, United States; b Department of Epidemiology and Biostatistics, Dornsife School of Public Health, Drexel, University. Nesbitt Hall, 3215 Market St., Philadelphia, PA 19104, United States; c Department of Health Systems and Population Health, School of, Public Health, University of Washington, 305 J Raitt Hall, Box 353410, Seattle, WA 98195, United States

**Keywords:** Supplemental nutrition assistance program, Immigrant origin, Policy, Immigration

## Abstract

**Introduction:**

Low-income immigrants who are eligible to participate in the Supplemental Nutrition Assistance Program (SNAP) participate at lower rates compared to non-immigrants. Immigrant households may be more likely to participate in SNAP if they live in areas with policies that integrate them into society and protect them from deportation.

**Methods:**

Data on low-income immigrant households came from the 2019 American Community Survey (*N* = 87,678). The outcome was whether any household member received SNAP in the previous 12 months. Immigrant policy exposures came from two sources: the State Immigration Policy Resource, which includes 18 immigrant criminalizing and integrating policies, and a database that identified ‘sanctuary policies’ (SP), which we summarized at the county level. Multivariable logistic regression adjusted for person/household-level and area-level confounders.

**Results:**

Living in a jurisdiction with a SP was associated with 21% higher odds of enrolling in SNAP compared to living in a jurisdiction without a SP (adjusted odds ratio [aOR] 1.21, 95% CI=1.11,1.31). Relative to the least immigrant friendly states, living in the most immigrant-friendly states was associated with 16% higher odds of SNAP enrollment (aOR=1.16, 95%CI=1.06–1.28). When SP and state-level immigrant friendly policy environment were cross-classified, SNAP participation was 23% and 26% higher for those living in jurisdictions with one- and both- exposures, respectively, relative to those with neither (aOR 1.23; CI 1.12,1.36; aOR 1.26; CI 1.15,1.37).

**Conclusions:**

Many at high risk of food insecurity – including immigrants and citizens in households with immigrants – are eligible for SNAP but under-enroll. Policies that welcome and safeguard immigrants could reduce under enrollment.

## Introduction

The Supplemental Nutrition Assistance Program (SNAP) is the largest nutrition assistance program in the U.S., providing a monthly allotment for food purchases to ∼43 million people in low-income households in 2020 (CBPP, 2022). SNAP participation is strongly associated with improved food security (Ratcliffe et al., 2011; Malbi and Ohls, 2015).

Approximately 14% of the US population are first generation immigrants (Budiman, 2020) and nearly one-fifth live in poverty (Huang et al., 2021). While undocumented immigrants are ineligible for SNAP, most legal immigrants who have lived in the U.S. for 5 years or more are eligible if they meet income requirements (USDA, 2011). However, eligible immigrants and those with close social ties to ineligible immigrants are less likely than non-immigrants to enroll in SNAP (Huang et al., 2021; Gelatt and Koball, 2014). In 2009, only 44% of eligible immigrant families enrolled in SNAP, compared to 65% of citizen-only families (Skinner, 2012). Immigrants face barriers to SNAP participation that are not experienced by U.S.born populations, including lack of program information and enrollment services that are culturally appropriate and in languages other than English (Gelatt and Koball, 2014).

State and local policies may also impact immigrants’ SNAP participation. Restrictive immigration policies and immigration enforcement activities can contribute to a general climate of fear and mistrust. This can lead immigrants to avoid public programs such as SNAP and interactions with public service providers out of fear of jeopardizing their residency status or that of others in their social network (Gelatt and Koball, 2014; Capps et al., 2020; Touw et al., 2021). The number of state-level bills related to immigrants and immigration increased four-fold from 2005 to 2009 (Skinner, 2012). Among the most relevant state and local policies relates to how law enforcement agencies participate in federal immigration enforcement. Numerous cities and counties have passed “sanctuary” policies that, in some way, affirm the rights of immigrants. Many such policies limit local/state law enforcement agencies’ cooperation with federal immigration authorities, particularly U.S. Immigration and Customs Enforcement (ICE). For example, sanctuary policies may prevent local authorities from complying with I-247 detention requests, which ask local authorities to detain undocumented immigrants until they can be released to ICE custody. States may also pass legislation that integrates immigrants into local communities, such as expanding eligibility for public programs and affirming eligibility of immigrants for other public services (e.g., public education, driver's licensing) (Broder and Lessard, 2023). Conversely, state and local law enforcement agencies and jails can enter into 287(g) program agreements with ICE that train and authorize local officers to perform specific functions related to the investigation, apprehension, or detention of immigrants (ICE, 2023).

The objective of this study is to investigate the effect of county- and state-level immigration-related policies on SNAP participation among immigrant households. We follow the analytic approach for health policy research where policies are explored at different levels of governance, which can provide a more comprehensive understanding of the impact of these policies (Schnake-Mahl et al., 2022). We posit that SNAP participation will be greater for individuals living in areas covered by sanctuary policies and in states with more immigrant integrating and fewer criminalizing policies.

## Methods

### Sample and sub-sample description

This cross-sectional study used individual-level data from the 2019 American Community Survey (ACS), accessed via IPUMS USA (Ruggles, 2023). ACS includes counties with populations of at least 65,000 residents (approximately 1% of the U.S. population); however, county-level geographic identifiers are only included for counties with at least 100,000 residents (approximately 60% of the ACS microdata). Broadly, the ACS microdata are constructed using a multi-stage design where housing units are selected to produce a sample generalizable to the U.S. population (Census Bureau, 2021).

**Fig. 1:**
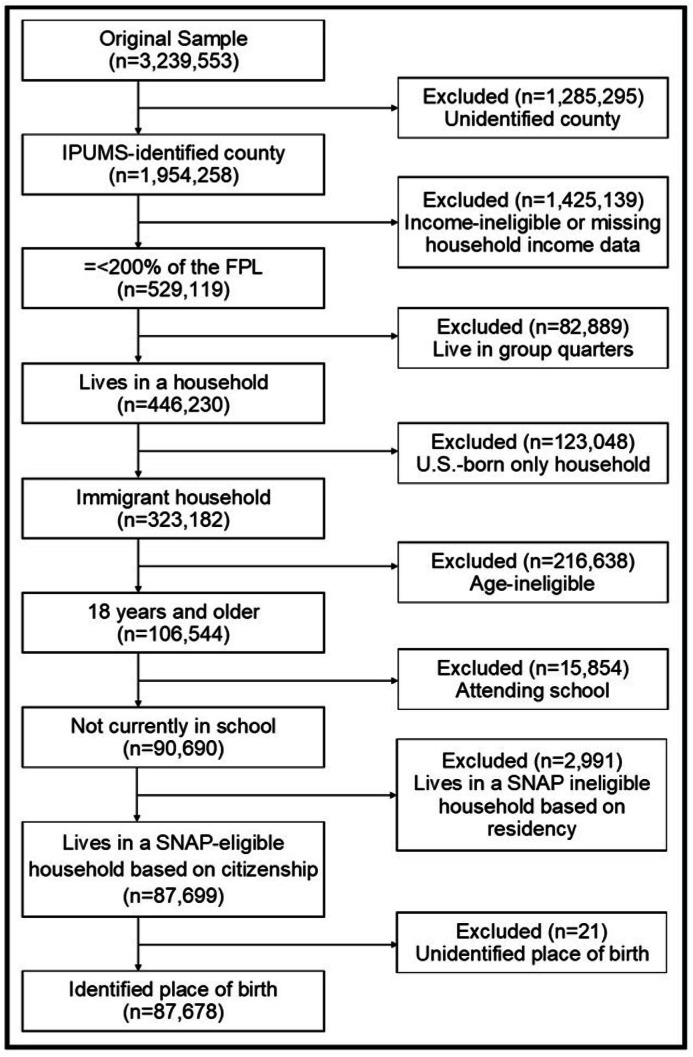
Flow chart showing exclusion of participants from the 2019 ACS sample by each exclusion criteria.

We defined our sample to include adults in households with at least one immigrant, in which at least one member of the household was likely eligible for SNAP. For the purposes of this study, an immigrant is a person who was born outside of the United States and outlying territories, irrespective of their documentation status. To approximate the sub-population eligible for SNAP, we excluded respondents based on the following criteria (see Fig. 1): 1) household income >200% of the federal poverty level (FPL); 2) residence in group quarters; 3) age <18 years, who typically do not make participation decisions for their households; 4) currently attending college; and 5) living in a household comprised exclusively of immigrants (i.e., foreign born individuals) who have lived in the U.S. for less than 5 years. We further excluded 21 immigrants for whom a country of origin was suppressed, because these data were used to develop a stratification variable for sensitivity analyses. Missing values of other variables are imputed by the Census Bureau. The final analytical sample included 87,678 participants.

### Measures

#### SNAP participation

The outcome variable is a dichotomous indicator of whether anyone in the household participated in SNAP within the previous 12 months.

#### Immigration policies

We used a database compiled by Lasch and colleagues (Lasch, 2018) that catalogued policy documents issued from 1979 to 2017 to identify states, counties, cities and towns that enacted immigrant integrating or supportive policies, broadly referred to as “sanctuary policies.” Policy documents affirmed the human rights of immigrants including prohibiting harassment and prohibiting local law enforcement from a specific type of activity (e.g., prohibition against sharing information about undocumented residents with ICE). Following prior work done by others (Ortiz et al., 2021; Chaparro et al., 2023), we classified county presence of a sanctuary policy if the county had ever passed a sanctuary policy itself, if the county is in a state that passed a sanctuary policy, or if a sanctuary policy had been passed in sub-county jurisdictions (i.e., cities) covering more than 50% of the county population.

We characterized **18 state-level immigrant-friendly policies** (see Appendix Table 1) using the Urban Institute State Immigration Policies database (Bernstein et al., 2023). This database compiled state-level immigrant policies in three domains: enforcement, public benefit access, and integration. They validated their conceptual framework by linking the state policy index to household-level data from the Survey of Income and Program Participation and confirming that the policies affected the material hardship of low-income immigrant households with children (J. Gelatt et al., 2023). Following indexing schemas for favorable and unfavorable policies developed by others (J. Gelatt et al., 2023; Monogan, 2013), we created a dichotomous variable for each policy, with a value of 0 if the policy was absent and either +1 or −1 if the policy was present and integrating or criminalizing, respectively. First, we averaged the means for policies in the public benefits, enforcement, and integration domains. Next, we averaged across the three domains to create a continuous, state-level ‘immigrant-friendliness score’ so that each domain has equal weight in the final score. Due to observed nonlinearity in the relationship between the policy score and household SNAP participation, we classified the state policy variable into tertitles which we subsequently labeled: unfriendly (bottom tertile, zero or less than 0.086), somewhat immigrant-friendly, and most immigrant-friendly (top tertile, 0.264 or greater). In sensitivity analyses results were substantively similar when we classified the state policy variable using quartiles (data not shown).

To assess the associations of SNAP participation with the **combined sanctuary policy** and **state immigrant-friendly policy environment**, we created a 3-level cross-classification variable using the binary sanctuary policy variable and a binary state immigrant-friendly policy environment. The 3-level variable represents: 1) both immigrant-friendly policy environments (sanctuary policy and most immigrant-friendly state group), 2) either (either sanctuary policy or most immigrant-friendly state group), and 3) neither (referent group). There were too few units in which the state was in the most friendly group, but the county was not covered by a sanctuary policy (20 counties within 3 states) to separate this into a standalone category.

#### Covariates

We selected confounders based on literature and a conceptual understanding of their impacts on the outcome of interest (SNAP participation) and where people reside (state/county immigrant policies). Individual-level covariates include age, sex, race/ethnicity, U.S. citizenship status (i.e., U.S.-born, naturalized, and non-citizen), educational attainment, and employment status. Household-level covariates include household nativity status where “immigrant only” households contain only foreign-born household members and “mixed status” households contain at least one foreign born and at least one U.S.born household member. Additional household characteristics are number of children in the household, % of the Federal Poverty Line (FPL), and linguistic isolation (defined as households where no one 14 years or over speaks English ‘very well’). County-level covariates include proportion of individuals over 65 and proportion with income below the FPL. Additionally, we adjusted for a summary measure of nine state policies that promote SNAP participation but do not specifically target immigrants (U.S. Department of Agriculture. Economic Research Service ERS, 1996; Klerman and Danielson, 2011).

### Statistical analyses

Frequencies, means, and standard errors of all variables were calculated, stratified by SNAP participation as well as by the 3-level combined county-state immigrant policy variable. We fit multivariable logistic regression models to assess the association between immigrant policy and SNAP participation. We progressively fit a series of three models for each of the three policy predictors: sanctuary policy, state immigrant policy, and the combined 3-category cross-classified immigrant policy variable (see Table 2 footnote for details).

SAS v9.4 (SAS Institute Inc., Cary, NC, USA) was used to compile the data and calculate descriptive statistics and the svy: logistic package in Stata MP 17.0 to fit multivariable logistic regression models. In all analyses, we used sampling weights and design variables to account for selection, non-response, and the complex sample design of ACS.

#### Sensitivity analyses

We conducted an exploratory sensitivity analysis to assess whether the association between the immigrant policy environment and SNAP participation varied by the household's region of origin (i.e., Latin American country, Muslim-majority country, non-Muslim-majority Asian country, and “other” countries – primarily those from Europe, Oceana, and non-Muslim African countries). We had no directional hypotheses for this analysis. The extreme political rhetoric that has demonized Latino, Muslim, and Asian immigrants in recent years could plausibly make these groups more sensitive to state and local immigrant integrating and criminalizing policies or, alternatively, could make these groups less responsive to state policy by reducing willingness to enroll among everyone regardless of state or local policies.

Since SNAP is allocated to households, we used individual respondent and household identifiers to identify the country of birth reported by non-U.S.born household members, and then assigned that country to a region of origin, and then subsequently to the household based on the following ordered hierarchy: 1) Latin American countries, 2) Muslim-majority countries, 3) Asian countries, 4) other countries in Europe, Oceania, and non-Muslim-majority African countries. Following work by others, Muslim majority refers to where at least 50% of the population is Muslim.

## Results

The majority of the sample consisted of individuals who identified as ‘Hispanic or Latino’ (58.6%, Table 1). Most of the sample lived in jurisdictions with sanctuary policies (72.5%) and with both sanctuary policies and immigrant-friendly states (55.6%). The proportion of the sample living in a household participating in SNAP ranged from 26.3% among those living in jurisdictions with a sanctuary policy and in an immigrant-friendly state to 23.7% among those living in jurisdictions with neither immigrant friendly policies.

**Table 1:** Characteristics of sample, stratified by SNAP participation and cross-classification of sanctuary and state immigrant policies.

		SNAP Participation		Cross-classification of sanctuary policy and state immigrant friendliness		
	Total	No	Yes	Both	Either	Neither
N	87,678	65,897	21,781	51,220	14,866	21,592
SNAP Participants, %	25.6	–	–	26.3	26.6	23.7
**Demographics**						
Age, Mean	47.4	47.4	47.6	48.8	46.8	45.0
Sex, %						
Male	45.4	46.8	41.4	45.1	45.5	45.9
Female	54.6	53.2	58.6	54.9	54.5	54.1
Race/Ethnicity, %						
Non-Hispanic White	15.7	16.3	14.1	15.4	14.3	17.4
Non-Hispanic Black	8.4	7.8	10.0	7.2	12.1	8.4
Non-Hispanic Asian	15.4	16.2	13.0	18.4	10.6	12.2
Non-Hispanic Other/Multiracial	1.9	1.9	2.0	2.0	1.8	1.8
Hispanic	58.6	57.8	60.8	57.0	61.2	60.1
**Nativity, Citizenship, & Language**						
U.S. Citizenship, %						
U.S.born citizen	20.8	21.0	20.4	19.2	22.9	22.9
Naturalized citizen	35.0	34.7	35.7	38.4	32.9	29.3
Not a citizen	44.2	44.3	43.9	42.4	44.2	47.9
Household nativity, %						
Mixed nativity	69.2	67.9	72.8	67.3	69.2	73.0
Immigrant only	30.8	32.1	27.2	32.7	30.8	27.0
Linguistically isolated, %	28.5	27.5	31.5	29.7	27.9	26.3
**Employment, education, & poverty**						
Unemployed (in labor force), %	4.5	4.2	5.4	4.8	4.4	3.8
Educational attainment, %						
No schooling	6.5	6.2	7.4	7.0	5.7	5.9
8th grade or less	17.3	16.5	19.6	18.0	14.9	17.5
Some HS	10.2	9.4	12.5	9.7	10.3	11.1
HS graduate	38.2	38.4	37.7	37.6	40.4	38.0
1–2 years of college	14.0	14.4	13.1	13.7	15.0	14.1
4 or more years of college	13.7	15.1	9.7	14.0	13.6	13.4
% of FPL, %						
1% or less of the FPL	5.9	6.2	5.1	6.0	6.2	5.6
2% to 49% of the FPL	8.4	7.1	12.0	8.3	8.1	8.8
50% to 99% of the FPL	22.8	19.4	32.6	23.2	22.4	22.0
100% to 149% of the FPL	29.7	29.9	29.1	29.5	30.1	29.9
150% to 200% of the FPL	33.2	37.3	21.2	33.0	33.3	33.6
**Household composition**						
Number of children in home, %						
0	46.6	49.0	39.9	48.5	48.7	41.3
1 or 2	34.5	34.6	34.2	33.9	34.3	35.9
3 or 4	16.5	14.8	21.4	15.5	15.1	19.7
5+	2.4	1.6	4.5	2.1	1.9	3.2
**Area-level characteristics**						
County % Adults aged 65+, mean	14.8	14.9	14.8	14.8	15.7	14.4
County % Individuals below the FPL, mean	13.0	12.7	14.1	12.6	13.4	13.6
County % Immigrants, mean	22.9	22.7	23.3	27.1	20.8	15.5
**Exposures: County & state policies**						
Sanctuary policy, %	72.5	71.8	74.5	–	–	–
State immigration policy, %						
Unfriendly	17.6	18.1	16.2	–	–	–
Somewhat friendly	25.7	25.8	25.7	–	–	–
Most friendly	56.6	56.1	58.1	–	–	–
Cross-classification of sanctuary policy and state “most friendly”, %						
Neither	26.5	27.2	24.4	–	–	–
Either	17.9	17.7	18.6	–	–	–
Both	55.6	55.1	57.0	–	–	–

After adjustment for individual- and household-level covariates, living in a jurisdiction **with a sanctuary policy** was associated with 21% higher odds of participating in SNAP compared to those not living in a jurisdiction with a sanctuary policy (Table 2). The association was nearly identical after adjustment for county- and state-level covariates (adjustment set 3: OR=1.21, 95%CI=1.11–1.31).

**Table 2:** Adjusted odds of SNAP participation by immigrant friendliness county and state policy exposures.

	*n* = 87,678	Unadjusted		Adjustment Set 1^a^		Adjustment Set 2^b^	
		OR [95% CI]		OR [95% CI]		OR [95% CI]	
**Policy A. Sanctuary policy**^c^**(not adjusted for policy B):**							
	No Sanctuary policy	Referent		Referent		Referent	
	Sanctuary policy	1.15^⁎⁎⁎^	[1.07,1.23]	1.19^⁎⁎⁎^	[1.11,1.28]	1.21^⁎⁎⁎^	[1.11,1.31]
**Policy B. State immigrant policy**^c^**(not adjusted for policy A):**							
	Unfriendly	Referent		Referent		Referent	
	Somewhat friendly	1.17^⁎⁎^	[1.064,1.282]	1.10*	[1.00,1.21]	1.01	[0.90,1.12]
	Most friendly	1.17^⁎⁎⁎^	[1.076,1.264]	1.15^⁎⁎^	[1.06,1.25]	1.16^⁎⁎^	[1.06,1.28]
**Combination of policies A and B. Cross-classification of sanctuary policy and state immigrant policy “most friendly”:**							
	Neither	Referent		Referent		Referent	
	Either	1.19^⁎⁎⁎^	[1.09,1.31]	1.24^⁎⁎⁎^	[1.13,1.36]	1.23^⁎⁎⁎^	[1.11,1.36]
	Both	1.14^⁎⁎⁎^	[1.07,1.23]	1.19^⁎⁎⁎^	[1.10,1.28]	1.26^⁎⁎⁎^	[1.15,1.37]

a Adjustment set 1 controls for age, sex, education, employment, income, and number of children in the household, race/ethnicity, household nativity, citizenship, and household linguistic isolation.

b Adjustment set 2 controls for all the variables in adjustment set 1 plus county-level percent unemployment, immigrant, adults over 65, and county urbanicity.

c 'Sanctuary policy' refers to living in a county that was subject to a sanctuary policy (Lasch et al. Understanding Sanctuary Cities database). 'Friendly state immigrant policy' refers to living in a state with favorable immigrant policies (Urban Institute's State Immigrant Policy Resource database). * *p* < 0.05, ** *p* < 0.01, *** *p* < 0.001.

**Table A1:** List of 18 state immigration policies and their coding scheme, grouped by domain and category.

			Coding scheme	
Domain	Category	Specific policies	Original	Revised
Integration	Education and driver's licenses	In-State Tuition	0,1	0, +1
		State Financial Aid	0,1	0, +1
		Ban from higher education	0,1	−1, 0
		English as official Language	0,1	−1, 0
		Driver's Licenses allowed for unauthorized immigrants	0,1	0, +1
Enforcement	Employment e-verify mandates	E-Verify mandates	0,1,2	−1, 0
		Prohibition on local E-Verify mandates	0,1	0, +1
Public Benefits	Cash benefits for LPRs	TANF for LPRs after 5-year bar	0,1	0, +1
		Cash assistance for LPRs during 5-year bar	0,1	0, +1
		Food assistance for LPR adults during 5-year bar	0,1	0, +1
		SSI replacement for LPRs	0,1	0, +1
Public Benefits	Health insurance for LPR and unauthorized	Medicaid for LPR children during 5-year bar	0,1	0, +1
		Public health insurance for LPR adults during 5-year bar	0,1	0, +1
		Medicaid for LPR pregnant women during 5-year bar	0,1	0, +1
		Medicaid for LPRs after 5-year bar	0,1	0, +1
		Public health insurance for unauthorized immigrant children	0,1	0, +1
		Public health insurance for unauthorized immigrant adults	0,1	0, +1
		Medicaid for unauthorized immigrant pregnant women	0,1	0, +1

LPR: Legal Permanent Residents are foreign-born individuals who have been granted the right to reside permanently in the United States.

Relative to living in an immigrant-unfriendly state, living in the most immigrant-friendly and somewhat friendly states was associated with 15% and 10% higher odds of SNAP participation, respectively (adjustment set 2: OR=1.15, 95%CI=1.06–1.25; OR=1.10, 95%CI= 1.00–1.21), after adjustment for individual and household covariates. However, after adjustment for county- and state-level covariates, the association was only sustained for participants living in a most immigrant-friendly state (OR=1.16, 95%CI=1.06–1.28).

When sanctuary policy and the state immigrant policy environment were cross-classified, the odds of SNAP participation were 23% and 26% higher for those living in jurisdictions with at least one of the conditions (OR=1.23; 95%CI=1.12–1.36) and both conditions present (OR=1.26; 95%CI=1.15–1.37), respectively, relative to those with neither. After adjustment for county- and state-level covariates, the association between areas with both conditions and SNAP participation strengthens, explained by a negative confounding effect of the area-level variables. This negative confounding effect is not present for the association between either condition and SNAP participation, which may be attributed to differences between areas.

**Fig. 2:**
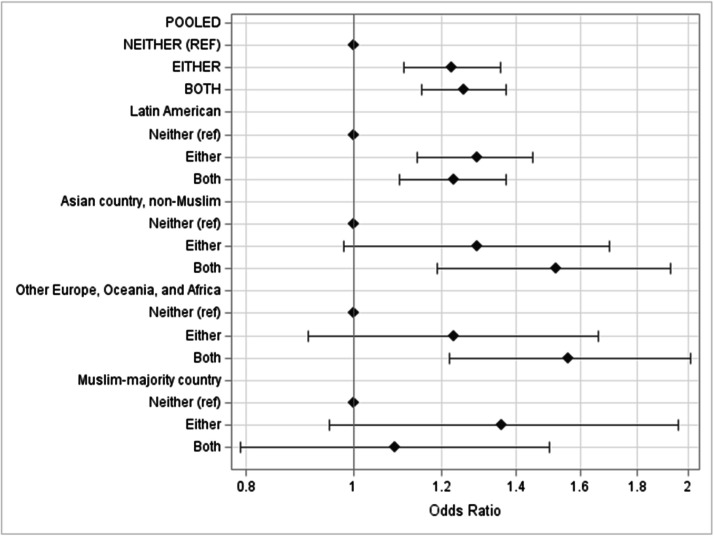
Plotted odds ratio of SNAP participation according to cross-classification of policies, stratified by region of origin. ^a^ Households were classified using a ordered hierarchy: 1) Latin American countries (65.4%), 2) else Muslim-majority countries (6.6%), 3) else Asian countries (15.6%), 4) else other countries in Europe, Oceania, and Africa (12.3%). Following work by others, Muslim majority refers to where at least 50% of the population is Muslim. b Models control for age, sex, education, employment, income, and number of children in the household, race/ethnicity, household nativity, citizenship, and household linguistic isolation as well as county-level percent unemployment, immigrant, adults over 65, and county urbanicity.

Fig. 2 and Appendix Table 2 show results from sensitivity analyses that explored whether the cross-classified policy variable and SNAP participation varied by household country/region of origin. These results suggest that, for households from all regions other than Muslim-majority countries, the associations between the policy variables and SNAP participation were qualitatively similar to the pooled results. Results appeared to differ for households with members coming from Muslim-majority countries; for these households, exploratory results suggested that living in jurisdictions with both types of policies did not have an effect on SNAP participation relative to living in jurisdictions with neither policy. However, the standard errors of these estimates were high, likely due to the small sample size among this sub-group.

## Discussion

In this cross-sectional study, we found that approximately 26% of people living in lower-income households with at least one immigrant (i.e., a household member born abroad) participated in SNAP within the past 12 months. Odds of SNAP participation were 21% higher for residents of areas with a sanctuary policy versus without a sanctuary policy; similarly, odds were 16% higher for those in states with the most-friendly versus the least-friendly immigrant policies. Further, odds of SNAP participation were at least 23% higher for those who lived in counties and states with either or both types of immigrant-friendly policies relative to those with neither.

Broadly, our results add to the emerging literature suggesting that immigrant integrating and criminalizing policies, as well as immigration enforcement actions, impact participation in public programs among both documented and undocumented immigrants, as well as those with close social ties to immigrants. This study complements recent work by Chaparro and colleagues, who found an ecological association between county and state immigration policies and county-level SNAP participation among Latino households (Chaparro et al., 2023). Alsan and Yang found that the roll-out of Secure Communities, a wide-ranging immigration-enforcement program created to help ICE arrest and remove individuals in violation of immigration laws by increasing information sharing between local and federal law enforcement agencies, was associated with reduced participation in federal means-tested programs among eligible Latino citizens that were not eligible for deportation (Alsan and Yang, 2022). This chilling effect was attenuated in areas with sanctuary policies, highlighting the importance of characterizing the local, state, and federal policies to which immigrants are exposed. Findings from a 2018 survey found that 15.6% of immigrants report that they or a family member avoided a noncash government benefit program, including SNAP, for fear of risking future residency eligibility (Bernstein et al., 2019). Hacker and colleagues conducted focus groups with documented and undocumented immigrants who reported stress and fear because of deportation risk, collaboration between local law enforcement and ICE, and needing to provide documentation (e.g., driver's license) to enroll in public insurance and health care programs (Hacker et al., 2011). Our findings strengthen this literature by suggesting that: 1) criminalizing policies likely cause eligible immigrants and those with close social ties to immigrants to forego participation in public programs, and 2) conversely, state and local sanctuary policies that reduce deportation risk and state integrating policies that provide immigrants with access to public benefits and services likely improve willingness to participate in SNAP.

Our sensitivity analyses suggest that the association between favorable immigrant policies and SNAP participation was qualitatively similar for immigrants from different regions of origin. The exception was for immigrant households from Muslim-majority countries. One explanation for divergent findings in this group may be that immigrant households from Muslim-majority countries are typically new arrivals and largely authorized immigrants (i.e., low levels of undocumented status) (Harjanto and Batalova, 2022). As a result, individuals in this group may be less likely to have undocumented immigrants in their households or to otherwise have close social ties to undocumented immigrants. This, in turn, may make immigrants from Muslim-majority countries less sensitive to state and local policies, particularly those that impact SNAP participation via increases in deportation risk that create a “culture of fear” (Berk and Schur, 2001; Hardy et al., 2012; Salas et al., 2013).

Collectively, our findings suggest that both passing immigrant-integrating policies and blocking or repealing immigrant-criminalizing policies is a potential pathway towards reducing the substantive levels of SNAP under participation in immigrant households. For example, our findings that SNAP participation was higher among immigrants in counties covered by sanctuary policies versus those without sanctuary policies is likely because sanctuary policies provide a buffer against federal immigration enforcement. Policymakers should also expand integrating policies that provide both documented and undocumented immigrants with access to programs and services that improve health (e.g., SNAP and Medicaid eligibility), as well as social and economic opportunities (e.g., driver's licenses, social services, public education). Examples of recent integrating policies include New York State's Excluded Workers Fund, which provided cash payments to workers who had been excluded from federal pandemic relief including undocumented immigrants (Waxman et al., 2023) and California using state funds to continue expanding eligibility for health and nutrition benefits to some undocumented immigrants (Gonzalez and Karpman, 2022). In addition, it is critical that databases of state and local integrating and criminalizing policies be periodically updated to document the spread of existing policies to new jurisdictions, and that databases be expanded to include new types of integrating and criminalizing policies. It is also important to catalogue both state and local policies, which are often discordant, particularly as states are increasingly passing preemption policies that reverse or restrict policies at the city or county level (Hall and Rhodes, 2020).

### Strengths and limitations

A major strength of this work is that we included multiple dimensions of immigrant policies observed at multiple geographies (state, county, sub-county). Analyzing immigration legislation at different levels of governance is needed to account for complexities in policy environments and the extent of residents’ exposure to policy implementation (Schnake-Mahl et al., 2022). A further strength of the study is its large sample size and geographic breadth as well as our ability to include an array of individual and area-level confounders.

As has been noted in many other studies of policy environments, our study was unable to assess neither the extent to which policies were actually implemented at the local level, nor households’ knowledge of the policies (Ortiz et al., 2021). However, prior qualitative work suggests that many immigrants are aware of local immigrant-criminalizing policies (Hacker et al., 2011). Despite the federal cut-off point for SNAP eligibility being <130% FPL gross income, the decision to include participants under 200% of the FPL was made to reflect states that have passed broad-based categorical eligibility policies. Using a cut-off of 130% income in analyses would have excluded SNAP-eligibles and participants in the most generous states. This has the limitation of also including individuals who may not be income-eligible for SNAP, depending on their state's SNAP policies. Lastly, SNAP participation was self-reported and the individual that completed the household component of the interview may not always be aware of program participation (Shantz and Fox, 2018). However, ACS is one of the few population-based data sources that samples across all U.S. states and that includes SNAP participation, immigration status, and other relevant individual- and household-level variables and has been used by many other researchers to examine participation in public programs (Skinner, 2012; Capps et al., 2020; Touw et al., 2021; Bernstein et al., 2019).

## Conclusion

The effects of immigrant policies on health-related outcomes remains an under-researched area, even though one out of seven U.S. residents is a first-generation immigrant (Budiman, 2020). Our study found immigrant households participate in SNAP in higher proportions when they are exposed to favorable immigrant policies at multiple levels, including state-level immigrant policies and sanctuary policies at the state, county, or sub-county level. The findings related to both sanctuary policies and state and local criminalizing policies suggest that federal policymakers should consider limiting the authority of ICE to delegate enforcement of federal immigration law to state and local officials. These findings extend beyond immigrant-related legislative policy, as the proposal of policies alone can create a climate of fear and a chilling effect among immigrants. More broadly, our findings imply that the individual and societal costs of food insecurity among immigrant households could be prevented by expanding policies that integrate immigrants into the social and economic life of U.S. society and that offer protection from threats of deportation (Gundersen and Ziliak, 2015).

## Funding

Research reported in this manuscript was in part supported by the United States Department of Health and Human Services National Institutes of Health (NIH) National Institute on Minority Health and Health Disparities under award number R01MD015107. The NIH played no role in the collection, analysis, interpretation or presentation of data. The content of this manuscript is solely the responsibility of the authors and does not necessarily represent the official views of the NIH.

## CRediT authorship contribution statement

**Sofia Argibay:** Writing – review & editing, Writing – original draft, Visualization, Validation, Project administration, Methodology, Formal analysis, Data curation, Conceptualization. **Amy H. Auchincloss:** Conceptualization, Methodology, Data curation, Writing – review & editing, Visualization. **M. Pia Chaparro:** Conceptualization, Methodology, Formal analysis, Writing – review & editing. **Caroline Kravitz:** Writing – review & editing, Methodology, Investigation, Conceptualization. **Alexandra Eastus:** Writing – review & editing, Methodology, Investigation, Conceptualization. **Brent A. Langellier:** Conceptualization, Methodology, Formal analysis, Writing – review & editing, Supervision, Funding acquisition.

## Declaration of competing interest

The authors declare they have no financial interests.

## Figures and Tables

**Table A2:** Odds Ratios of SNAP participation according to cross-classification of policies, stratified by household (HH) place of origin.

	Latin American country HH		Muslim-majority country HH		Asian^a^ country HH		Other country HH	
Neither		Referent		Referent		Referent		Referent
Either	1.29^⁎⁎⁎^	[1.14,1.45]	1.36	[0.95,1.96]	1.29	[0.98,1.70]	1.23	[0.91,1.66]
Both	1.23^⁎⁎⁎^	[1.10,1.37]	1.09	[0.79,1.50]	1.52^⁎⁎^	[1.19,1.93]	1.56^⁎⁎⁎^	[1.22,2.01]

^a^Non-muslim majority Asian countries.

^b^Adjustment set controls for age, sex, education, employment, income, and number of children in the household, race/ethnicity, household nativity, citizenship, household linguistic isolation, county-level percent immigrant, unemployment, adults over 65, and county urbanicity.

Exponentiated coefficients; 95% confidence intervals in brackets.

* *p* < 0.05, ** *p* < 0.01, *** *p* < 0.001.
